# A mouse model of *Citrobacter rodentium* oral infection and evaluation of innate and adaptive immune responses

**DOI:** 10.1016/j.xpro.2020.100218

**Published:** 2020-12-14

**Authors:** Wenyan Wang, Yiping Li, Xiaohuan Guo

**Affiliations:** 1 Institute for Immunology, Tsinghua University, Beijing 100084, China; 2 Department of Basic Medical Sciences, School of Medicine, Tsinghua University, Beijing 100084, China; 3 Beijing Key Lab for Immunological Research on Chronic Diseases, Tsinghua University, Beijing 100084, China

**Keywords:** Cell Isolation, Immunology, Model Organisms

## Abstract

*Citrobacter rodentium* is an extracellular enteric bacterial pathogen that induces both innate and adaptive immunity in mice, its natural host. Here, we detail the step-by-step procedure to evaluate the immune responses in a mouse model of *C. rodentium* infection. We describe the methods to establish infection, isolate group 3 innate lymphoid cells from lamina propria lymphocytes, and analyze their response. We also assess the response of T follicular helper cells and germinal center B cells.

For complete details on the use and execution of this protocol, please refer to Guo et al. (2015), Kennedy and Hartland, (2018), and Wang et al. (2020).

## Highlights

• A protocol for the mouse model of *C. rodentium* infection
• Procedure for evaluation of *C. rodentium* burden in the feces post infection
• Isolation of LPLs from the gut and assessment of ILC3s post *C. rodentium* infection
• Assessment of TFH and GCB responses post *C. rodentium* infection

## Graphical Abstract

**Figure:**
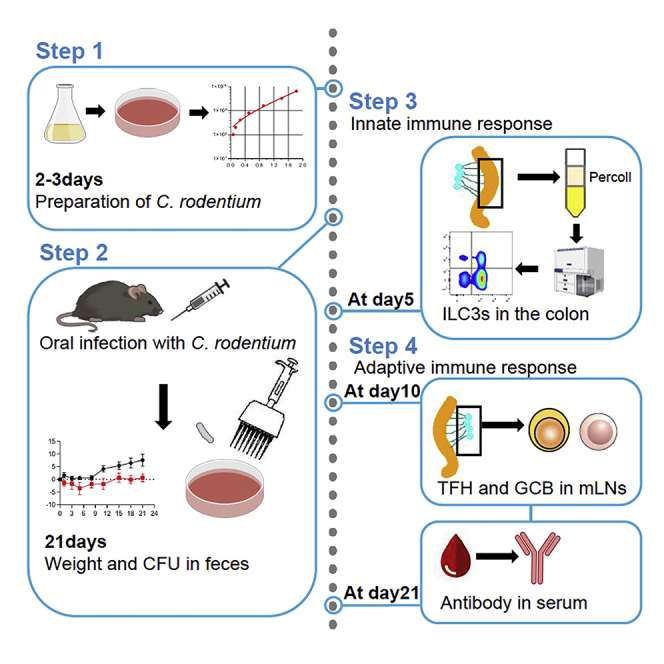

## Before you begin

### Standard growth curve of *Citrobacter rodentium* preparation

**Timing: 2.5 days**

1. Inoculate *Citrobacter rodentium* (*C. rodentium*) in 4 mL LB broth, and culture it for 16 h in 37°C, 200 rpm in a shaker incubator.
2. Measure the concentration of *C. rodentium* by spectrophotometer at 600 nm of optical density (OD600).
  a) Prepare seven 5-mL tubes and label as C7, C6, C5, C4, C3, C2, C1, respectively (as shown in Figure 1A).

**Figure 1:**
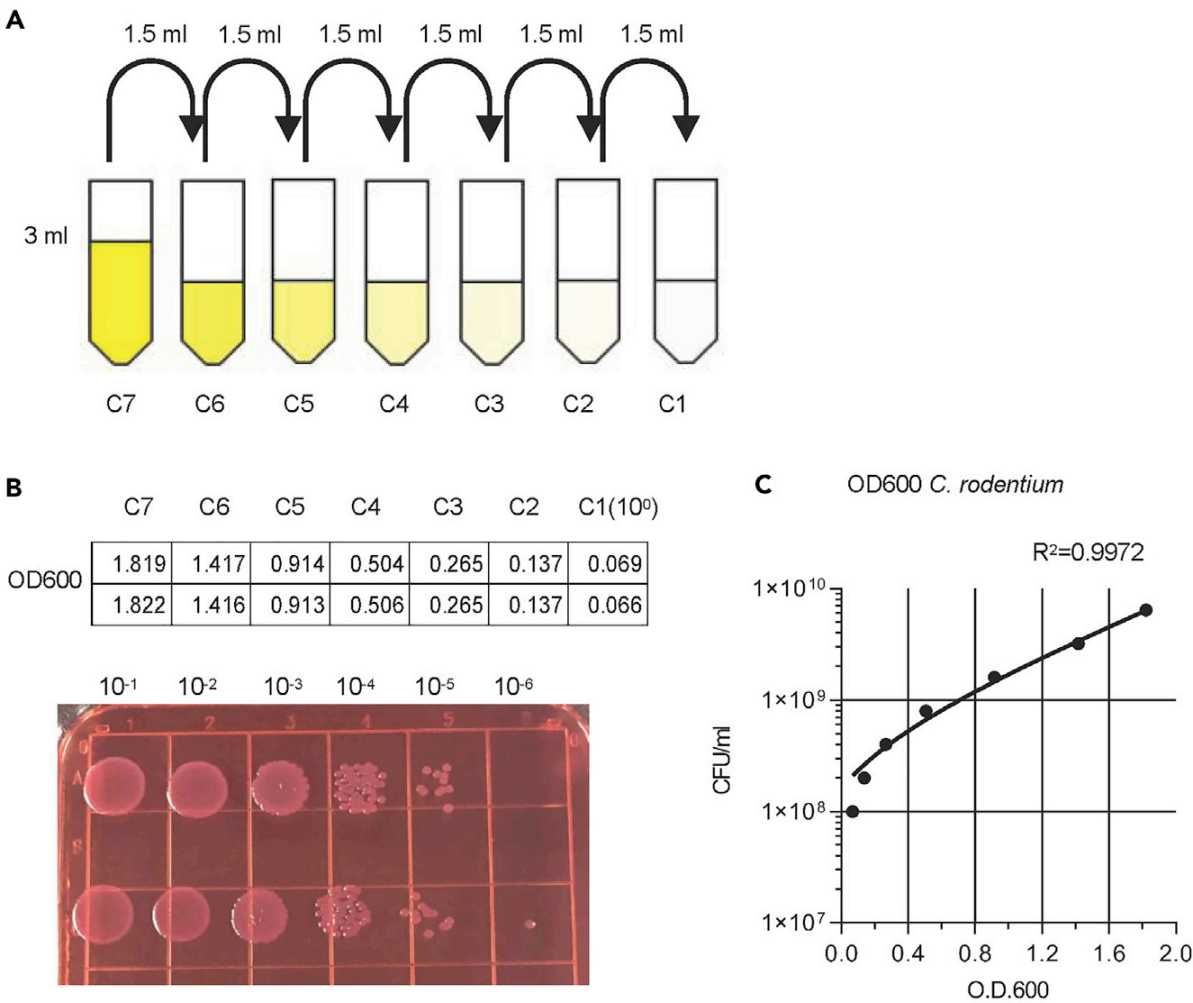
The preparation of the standard curve for measuring *C*. *rodentium* CFU (A) The diagram for the dilution of *C*. *rodentium*. (B) The value of OD600 for each dilution and CFU of dots on the plate. (C) The example standard curve.
  b) Add 3 mL of bacteria medium to C7 tube as the top concentration, and add 1.5 mL LB broth to C6-C1 tubes.
  c) Transfer 1.5 mL of top standard C7 to C6 and mix by pipetting.
  d) In the same manner, dilute at 1:2 ratio from concentration C6 to C1, one by one.
  e) Take LB broth as blank, detect OD600 of standard C7-C1, by the spectrophotometer.
3. Measure the concentration of *C. rodentium* by counting the colony forming units (CFU) on the MacConkey Agar plate.
  a) Prepare 6 microcentrifuge tubes and label as 10^−1^, 10^−2^, 10^−3^, 10^−4^, 10^−5^, 10^−6^, respectively. Relabel standard C1 as 10^0^.
  b) Add 180 μL LB broth to 10^−1^–10^−6^ tubes, transfer 20 μL of 10^0^ to 10^−1^ tube and mix wells by pipetting.
  c) In the same manner, dilute standard 10^0^ at 1:10 ration from 10^−1^ to 10^−6^, one by one. Duplicate each dilute.
  d) Dot 10 μL of every standard on the MacConkey Agar plate, with proper spacing.
  e) Culture the plate in the 37°C incubator for 12–16 h.
  f) Count the CFU of the last two countable dots on the plate (as shown in Figure 1B). The ratio of two adjacent dots should be approximately 10.
4. Draw standard growth curve of *C. rodentium.*
  a) Calculate the concentration of C7-C1, according to the dilution ratio.
  b) Statistically analyze the correlation of CFU and OD600 of standard C7-C1, to visualize the curve of *C. rodentium* growth (as shown in Figure 1C).

**CRITICAL:** 1. Because the concentration of bacteria is too high to count on the plate even in C1 group, continue to dilute C1-C7 as 1:10 and dot on the MacConkey Agar plates to calculate the concentration of bacteria. 2. Fewer than 20 colonies of one dilution is considered as countable on the plate.

### *Citrobacter rodentium* inoculum preparation

**Timing: 2 days**

5. Streak *C. rodentium* on a MacConkey Agar plate, and culture it in the 37°C incubator for 12–16 h.
6. Pick a single colony and inoculate it in 2 mL LB broth, culture for 16 h in 37°C, 200 rpm in a shaker incubator.
7. Calculate the CFU of bacteria.
  a) Measure OD600 of the *C. rodentium* culture medium.
  b) Calculate the CFU according to the standard growth curve.
  c) Harvest enough *C. rodentium* at 3,500 × *g* in 4°C for 8 min.
  d) Discard the supernatant and resuspend the pellet with sterile PBS buffer at a proper concentration.

If the infection dose is 2 × 10^9^ CFU/200 μL PBS per mouse, resuspend the bacteria at 1 × 10^10^ CFU/mL PBS. If the infection dose is 5 × 10^6^ CFU/200 μL PBS per mouse, resuspend the bacteria at 2.5 × 10^7^ CFU/mL PBS. If 10 mice are required to be infected, prepare enough *C. rodentium* at least for 15 mice to prevent potential loss. For example, if the OD600 of *C. rodentium* culture medium is 1.600, the concentration will be 4.5 × 10^9^ CFU/mL. If 10 mice are required to be infected at the dose of 5 × 10^6^ CFU/200 μL PBS per mouse, dilute the *C. rodentium* culture medium 10-fold and take 167 μL of medium to spin down, then resuspend the bacteria with 3 mL PBS. The *C. rodentium* medium will be at a concentration of 2.5 × 10^7^ CFU/mL and enough for 15 mice (200 μL/mouse).

8. Confirm the concentration of gavaged *C. rodentium* by serially diluting the culture and the colony forming units on the MacConkey Agar plate.

**CRITICAL:** 1. The concentration of *C. rodentium* used for infection is dependent on the purpose of experiments (Guo et al., 2015). A high dose of 10^8^–10^9^ CFU is usually used in other protocols (Bouladoux et al, 2017) to study the pathogenesis of *C. rodentium* or innate/adaptive immune responses. Here we used a low dose of *C. rodentium* in order to observe the dynamic colonization of the pathogen in the early stage and examine the earlier innate immune response. While assessing the adaptive immune response, a dose of 2 × 10^9^ CFU per mouse was used. 2. Make sure that the total volume of PBS to resuspend the bacteria will not exceed 300 μL per mouse.

### Antibody mix preparation

**Timing: 1 h**

9. Make antibody mix for staining group 3 innate lymphoid cells (ILC3s) as Antibody Mixture 1, 2, 3.
  a) Antibody Mixture 1:
  0.1 μL of FITC-CD3, Pacific Blue -CD45, Brilliant Violet 510-CD90, Brilliant Violet 605-CD4; 0.05 μL of eFluor-780 and 0.15 μL of PE/Cyanine7-CD127 in 50 μL FACS buffer/sample.
  b) Antibody Mixture 2:
  0.2 μL of PerCP-eFluor 710-RORγt in 50 μL/sample permeabilization buffer.
  c) Antibody Mixture 3:
  0.2 μL of PerCP-eFluor 710-IL22 in 50 μL/sample permeabilization buffer.
10. Make antibody mix for staining germinal center B cell (GCB) and T follicular helper cell (TFH) as Antibody Mixture 4, 5.
  a) Antibody Mixture 4:
  0.1 μL of Alexa Fluor 700-CD45, Pacific Blue-B220, 0.15 μL of Alexa Fluor 647-GL7, PE-CD95, and 0.05 μL of eFlour-780 in 50 μL/sample FACS buffer.
  b) Antibody Mixture 5:
  0.1 μL of Alexa Fluor 700-CD45, PE/Cyanine7-CD3, APC/Cyanine7-CD4, APC Streptavidin, 0.15 μL of FITC-CD44, PerCP-PD-1, and 0.05 μL of eFluor-506 in 50 μL/sample FACS buffer.

## Key resources table

**Table:** 

REAGENT or RESOURCE	SOURCE	IDENTIFIER
**Antibodies**		

Pacific Blue anti-mouse CD45	Biolegend	AB_493535
PE/Cyanine7 anti-mouse CD127	Biolegend	AB_1937265
Alexa Fluor 647 anti-MU/HU GL7 antigen	Biolegend	AB_2562185
Pacific Blue anti-mouse/human CD45R/B220	Biolegend	AB_492876
Alexa Fluor 700 anti-mouse CD45	Biolegend	AB_493715
Biotin anti-mouse CD185 (CXCR5)	Biolegend	AB_2562126
PerCP/Cyanine5.5 anti-mouse CD279 (PD-1)	Biolegend	AB_2159184
FITC anti-mouse/human CD44	Biolegend	AB_312956
APC/Cyanine7 anti-mouse CD4	Biolegend	AB_312699
PE anti-CD95	BD Bioscience	AB_10895586
Brilliant Violet 605 anti-mouse CD4	eBioscience	AB_2564591
FITC anti-mouse CD3e	Biolegend	AB_312671
PE/Cyanine7 anti-mouse CD3e	Biolegend	AB_312685
Brilliant Violet 510 anti-mouse CD90.2	Biolegend	AB_2561395
IL-22 monoclonal antibody (1H8PWSR), PerCP-eFluor 710	eBioscience	AB_10598646
APC streptavidin	Biolegend	Cat# 405207
ROR gamma (t) monoclonal antibody (B2D), PerCP-eFluor 710	eBioscience	AB_10717956
eFluor-780	eBioscience	Cat# 65–0865-14
eFluor-506	eBioscience	Cat# 65-0866-18
Fixation concentrate	Thermo Fisher Scientific	Cat# 00-5123-43
Fixation/perm diluent	Thermo Fisher Scientific	Cat# 00-5223-56
Permeabilization buffer	Thermo Fisher Scientific	Cat# 00-8333-56
IC fixation buffer	Thermo Fisher Scientific	Cat# 00-8222-49
Brefeldin A solution (1,000×) BFA	Biolegend	Cat# 420601
Penicillin-streptomycin	Thermo Fisher Scientific	Cat# 15140163
Phorbol 12-myristate 13-acetate (PMA)	Sigma-Aldrich	Cat# P8139
Ionomycin calcium salt	Tocris	Cat# 1704/1
HBSS (10×), calcium, magnesium, no phenol red	Gibco	Cat# 14065056
HBSS (10×), no calcium, no magnesium, no phenol red	Gibco	Cat# 14185052
HEPES (1 M)	Gibco	Cat# 15630130
UltraPure 0.5 M EDTA	Thermo Fisher Scientific	Cat# 15575020
Percoll	GE Healthcare	Cat# 17-0891-01

**Bacterial and virus strains**		

*Citrobacter rodentium* DBS100	ATCC	Cat# 51459

**Chemicals, peptides, and recombinant proteins**		

DNase I	Sigma	Cat# D5025
Liberase TL	Roche	Cat# 5401046001

**Critical commercial assays**		

B-PER bacterial protein extraction reagent	Thermo Fisher Scientific	Cat# 78243
Pierce BCA protein assay kit	Thermo Fisher Scientific	Cat# 23225
SBA Clonotyping System-B6/C57J-HRP	SouthernBiotech	Cat# 5300-05B

**Experimental models: mouse model**		

C57BL/6 mouse	Tsinghua University	N/A

**Software and algorithms**		

GraphPad Prism 7.0	GraphPad	https://www.graphpad.com/
FlowJo 10	FlowJo	https://www.flowjo.com/

## Materials and equipment

### Wash buffer I

**CRITICAL:** The Wash Buffer I should be prepared freshly before application, stored in 4°C.

**Table:** 

Reagent	Final concentration	Amount
10× HBSS without Ca^2+^ and Mg^2+^		10 mL
HEPES buffer	10 mM	1 mL
0.5 M EDTA	5 mM	1 mL
Dithiothreitol (DTT)	1 mM	15.425 mg
FBS	3%	3 mL
ddH_2_O		87 mL
**Total**		**100 mL**

**Table:** Wash buffer II

Reagent	Final concentration	Amount
10× HBSS without Ca^2+^ and Mg^2+^		10 mL
HEPES buffer	10 mM	1 mL
ddH_2_O		89 mL
**Total**		**100 mL**

### Digestion buffer

**CRITICAL:** The Digestion Buffer should be prepared freshly before application, stored in 4°C.

**Table:** 

Reagent	Final concentration	Amount
HEPES buffer	10 mM	1 mL
FBS	3%	3 mL
100 mM penicillin-streptomycin	1 mM	1 mL
Liberase TL	0.1 mg/mL	10 mg
DNase I	0.05%	50 mg
RPMI-1640		95 mL
**Total**		**100 mL**

**Table:** 1640 buffer

Reagent	Final concentration	Amount
HEPES buffer	10 mM	1 mL
FBS	3%	3 mL
100 mM penicillin-streptomycin	1 mM	1 mL
RPMI-1640		95 mL
**Total**		**100 mL**

**Table:** 100% Percoll

Reagent	Final concentration	Amount
10× HBSS with Ca^2+^ and Mg^2+^		10 mL
HEPES buffer	10 mM	1 mL
100 mM penicillin-streptomycin	1 mM	1 mL
Percoll		88 mL
**Total**		**100 mL**

**Table:** DMEM buffer

Reagent	Final concentration	Amount
HEPES buffer	10 mM	1 mL
FBS	3%	3 mL
100 mM penicillin-streptomycin	1 mM	1 mL
DMEM		95 mL
**Total**		**100 mL**

**Table:** FACS buffer

Reagent	Final concentration	Amount
Sodium azide (NaN_3_)	0.02%	20 mg
FBS	2%	2 mL
PBS buffer		98 mL
**Total**		**100 mL**

80% Percoll: 20 mL PBS buffer+80 mL 100% Percoll.

Coating buffer: 15 mM Na_2_CO_3_, 35 mM NaHCO_3_, pH 9.6 in H_2_O.

PBST buffer: PBS buffer+0.5% Tween20.

### Mice

All mice were on C57BL/6 background. The mice were raised and maintained under specific pathogen-free conditions at Tsinghua University. All studies were approved by the Animal Care and Use Committee of Tsinghua University. The diet was purchased from Jiangsu Xietong pharmaceutical Bioengineering Co., Ltd (Cat# 1010008), which was used for mice growth and reproduction.

## Step-by-step method details

### Oral infection of mice with *Citrobacter rodentium*

**Timing: 21 days**

1. One day pre-infection (day −1).
  a) 8–9 weeks old mice are chosen for infection.
  b) Record the weight of mice.
  c) Fast the mice for 16 h with water given ad libitum before infection.
2. At day 0.
  a) Orally gavage the mice with 5 × 10^6^ CFU of *C. rodentium* in 200 μL PBS using the gavage needle (8#) (as shown in Figure 2A).

**Figure 2:**
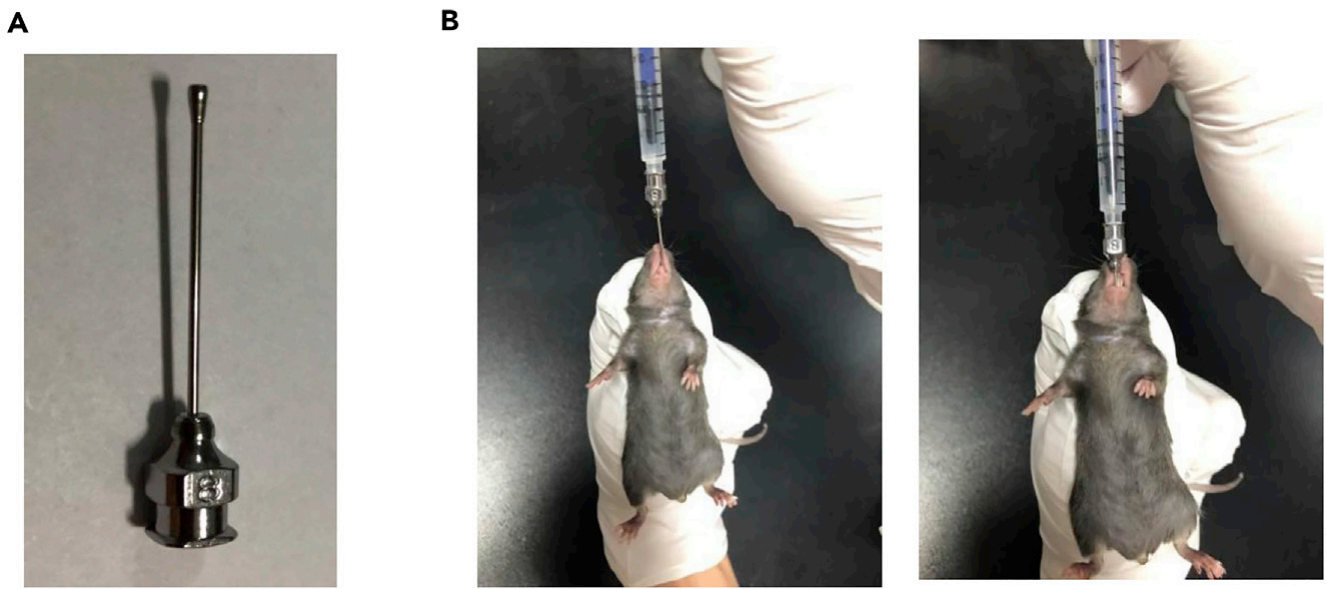
Oral inoculation with *C. rodentium* (A) The gavage needle. (B) The procedure of oral gavage.
  b) Insert the needle parallel to the upper jaw until the throat. Verticalize the needle and insert along the esophagus without any obstruction, until almost the whole needle is inserted (as shown in Figure 2B).
  c) Inject 200 μL of bacteria gently with no liquid overflowed from the mouth.
  d) Resume the diet.
3. At day 1 to day 21.
  a) Record the weight of mice every day, analyze the weight change according to the weight at day −1. See Troubleshooting 1.
  b) Observe the stool consistency and recorded as the scoring criteria (0: normal, 1: loose stool, 2: shapeless loose stool, 3: diarrhea, 4: diarrhea with bleeding).
  c) Collect feces into sterile microcentrifuge tubes every other day. Detect the CFU of *C. rodentium* in the feces as the protocol of “Determine the *C. rodentium* CFU in the feces".
  d) Detect the ILC3s response at day 5 as the protocol of “Assessment of the ILC3s response".
  e) Detect the adaptive T cell response at day 10 and the antibody response at day 21 as the protocol of “Assessment of the adaptive humoral immune response".

**CRITICAL:** 1. The gavage needle is stainless steel in material, 45 mm in length, and straight. 2. To make sure that the feces could be collected, avoid frightening the mice by moving the cage or making noise before the collection. 3. It is better to fetch the feces directly by attaching the microcentrifuge tube against the anus. When infected at a high dose or around day 5–7 post infection, the feces may be difficult to collect because of the dysentery. Watery stools can be scraped into the tube and estimate the volume instead of the weight.

### Determine the *C. rodentium* CFU in the feces

**Timing: 1 h**

**Figure 3:**
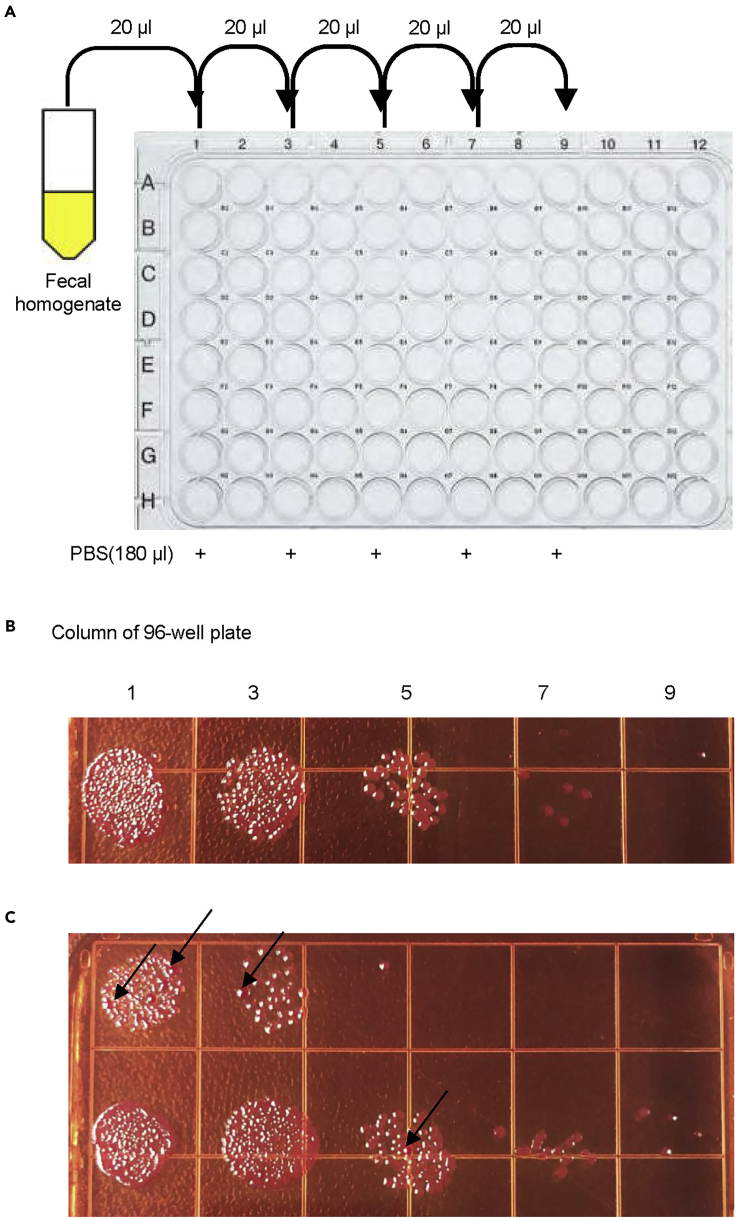
CFU counting of *C. rodentium* in the feces (A and B) The model for the dilution of fecal homogenate (A) and CFU of *C. rodentium* dot on the MacConkey Agar plate in 10-fold dilution gradient (B). (C) Other bacteria colonies like *E. coli* showing on the plate (black arrow).
  e) Incubate the plate in 37°C for 12–16 h.
  f) Count CFU of the last two countable dots on the plate (as shown in Figure 3B). See Troubleshooting 2.
  g) Calculate the concentration of *C. rodentium* in the feces according to the formula:

4. Make normalized fecal homogenate.
  a) Weight the feces using an analytical balance and transfer it to a sterile 5 mL tube.
  b) Add 1 mL PBS buffer to every 50 mg feces.
  c) Homogenize the samples thoroughly using a tissue grinder.
5. Count the CFU of *C. rodentium* in the feces.
  a) Prepare a U-bottom 96-well plate, 2 multichannel pipettes, and PBS buffer.
  b) Add 180 μL PBS to interlaced column of the 96 well plate.
  c) Add 20 μL of the sample to the first column. Mix with pipettes and transfer 20 μL to the next column, and so on.
  d) For each sample, dot 10 μL of each well by the multichannel pipette with interlaced tips on the MacConkey Agar plate (as shown in Figure 3A).

C (CFU/g) = CFU in the dot × 2 × 10^(n+3)^, n= the order of the dot counted. Take the samples shown in Figure 3B, the CFU=5 × 2 × 10^(4+3)^ = 1 × 10^8^ CFU/g.

**CRITICAL:** 1. In step 1, wash the tissue grinder with ethanol twice and water twice between samples. In step 2, the data will be reliable if the ratio of two adjacent dots is approximately 10. 2. Other bacteria colonies should be excluded (as shown in Figure 3C).

### Assessment of the ILC3s response

**Timing: 5 h**

**Figure 4:**
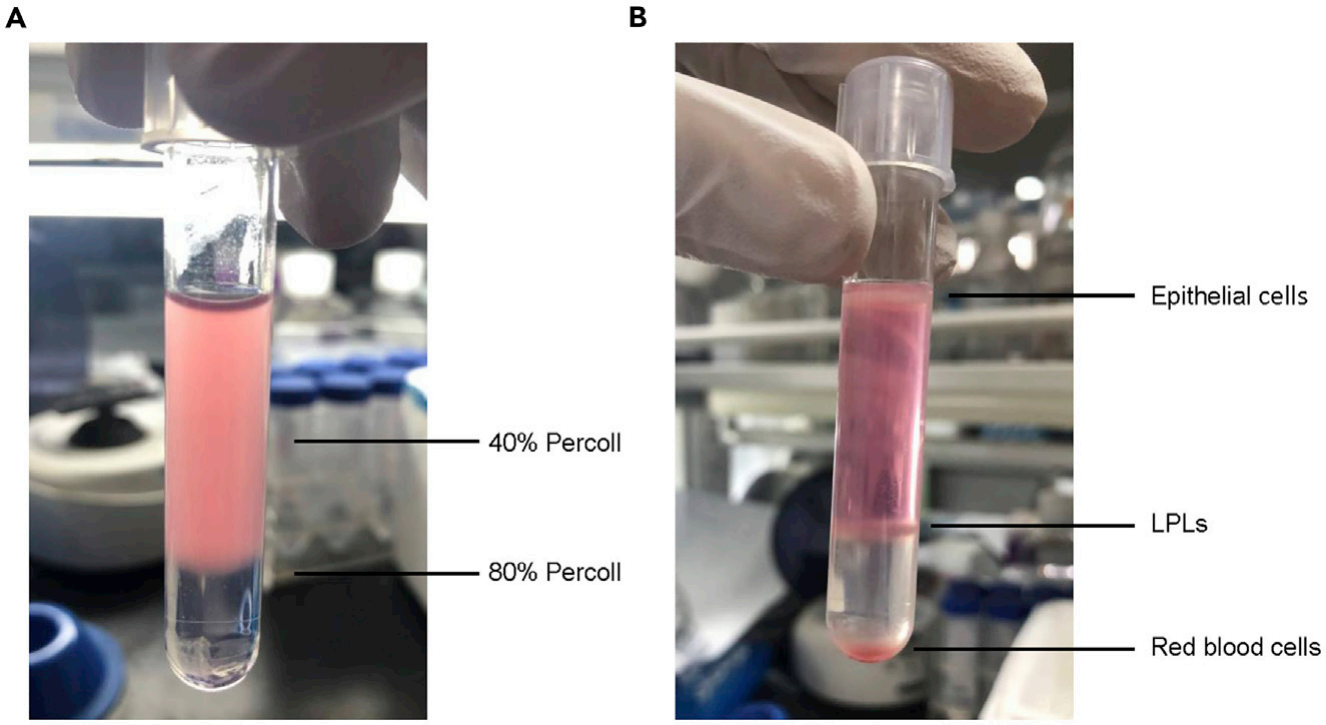
Isolation of LPLs by Percoll The LPLs isolation from the colon before (A) and after (B) the centrifugation with Percoll.
  k) Carefully remove the top layer of epithelial cells and upper 40% Percoll, collect the LPLs with the micropipette into a 5 mL tube.
  l) Wash the cells with 4 mL FACS buffer by centrifuging at 4°C 650 × *g* for 5 min, twice. Discard the supernatant and resuspend the cells with 1 mL FACS buffer.
  m) Mix 10 μL 0.4% Trypan Blue with 10 μL cells mixture, and count the unstained cells (living cells) with the hemocytometer. See Troubleshooting 3 and 4.

6. Isolation of the lamina propria lymphocytes (LPLs) from the colon (Guo et al., 2016).
  a) At day 5 post infection, euthanize the mice by cervical vertebra dislocation and isolate the colon. Remove the content in the gut thoroughly by scraping, sliver the colon, and cut the tissues into 0.5 cm pieces.
  b) Wash the tissues with 20 mL PBS in a 50 mL centrifuge tube by vertexing to clean up the content remained and transfer the tissues to Wash Buffer I.
  c) Wash the tissues with 15 mL Wash Buffer I in 37°C, shake at 200 rpm on a shaking table for 20 min, twice.
  d) Wash the tissues with 15 mL Wash Buffer II in 37°C, shake at 200 rpm on a shaking table for 20 min.
  e) Digest the tissues in 2.5 mL digestion buffer in a C-tube in 37°C, shake at 150 rpm on a shaking table for 30 min.
  f) Homogenize the tissues with the gentleMACS Dissociator for 1 minute and add 10 mL 1640 buffer to stop the digestion.
  g) Apply the samples to the 70 μm cell filter to make single cell suspension.
  h) Centrifuge the samples in 4°C, 650 × *g* for 5 min, resuspend the cells with 1 mL DMEM buffer and mix with 1 mL 80% Percoll solution (to make 40% Percoll solution) in a 5 mL tube.
  i) Carefully add 1 mL 80% Percoll solution at the bottom of the suspension. The clear separation of layers should be observed (as shown in Figure 4A).
  j) Centrifuge the samples in 4°C, 950 × *g* with no brake for 20 min. The cell cloud between 40% Percoll and 80% Percoll is the LPLs (as shown in Figure 4B).

**CRITICAL:** 1. Store the tissues in PBS on the ice until the Wash Buffer I. 2. If the gentleMACS Dissociator is not available, cutting the tissue until homogenized by scissors is an option. In that case, the cutting step should be done before the digestion. 3. The viability of LPLs should be over 90% for further analysis. The average number of LPLs is usually about 1 × 10^6^–5 × 10^6^ per colon.

For all wash steps that are not mentioned specifically in this section, cells should be centrifuged at 4°C 650 × *g* for 5 min.

7. Examine ILC3 in the LPLs.
  a) For quantifying ILC3s:
    i. Resuspend 2 × 10^6^ LPLs with 50 μL Blocking buffer to block Fc receptor internalization.
    ii. For staining ILC3s, incubate the LPLs with Antibody Mixture 1 in 4°C for 30 min.
    iii. Wash the cells with FACS buffer.
    iv. Fix the cells with 100 μL fixation buffer at room temperature (20°C–25°C) for 20 min.
    v. Wash the cells with 200 μL permeabilization buffer at room temperature (20°C–25°C) for 3 times.
    vi. Incubate the cells with Antibody Mixture 2 in room temperature (20°C–25°C) for 30 min.
  b) For function of ILC3s:
    i. Resuspend 2 × 10^6^ LPLs with 200 μL 1640 medium with 10% FBS, 10 mM HEPES and 1 mM Penicillin-Streptomycin and incubate at 37°C with 5% CO_2_. Stimulate with PMA (20 ng/mL) and Ionomycin (750 ng/mL) for 2 h, and add BFA for another 2 h.
    ii. Wash the cells with FACS buffer and incubate the LPLs with Antibody Mixture 1 in 4°C for 30 min.
    iii. Wash the cells with FACS buffer.
    iv. Fix the cells with the IC fixation buffer at 4°C for at least 12 h.
    v. Wash the cells with the permeabilization buffer at room temperature (20°C–25°C) for 3 times.
    vi. Incubate the cells with Antibody Mixture 3 in room temperature (20°C–25°C) for 30 min.
  c) Wash and then resuspend the cells with FACS buffer, and detect the ILCs by flow cytometry.
  d) Firstly, define lymphocytes by gating CD45^+^ population and exclude dead cells by gating V780^−^ cells. In the colon, ILCs can be gated as CD3^−^CD90^+^CD127^+^. In ILCs, RORγt^+^ cells are defined as ILC3s. See Troubleshooting 5.

### Assessment of the adaptive humoral immune response

**Timing: 4.5 h**

For all wash steps in this section, cells should be centrifuged at 4°C, 650 × *g* for 5 min.

8. Detection of GCB and TFH response in the mesentery lymph nodes (mLNs) (Melo-Gonzalez et al., 2019). In mLNs, the germinal center is the place where B cells become mature in stimulation, which could be regulated by TFH cells. TFH and GCB can promote the production of high affinity antibodies to maintain intestinal homeostasis (Hepworth et al., 2015).
  a) At day 10 post infection, euthanize the mice by cervical vertebra dislocation and isolate the mLNs (as shown in Figure 7A).
  b) Grind the mLNs to make single cell suspension.
  c) Resuspend 2 × 10^6^ lymphocytes with Blocking buffer to block Fc receptor internalization.
  d) To stain GCB, incubate the lymphocytes with Antibody Mixture 4 in 4°C for 30 min.
  e) To stain TFH, incubate the lymphocytes with biotin-labeled CXCR5 antibody in 4°C for 30 min.
  f) Wash the cells FACS buffer in 4°C for twice, and then incubate the cells with Antibody Mixture 5 in 4°C for 30 min.
  g) Wash and then resuspend the cells with FACS buffer, and detect the lymphocytes by flow cytometry.
9. Analysis of *C. rodentium*-specific antibodies in the serum (Wang et al., 2020).
  a) At day 21 post infection, collect the peripheral blood from epicanthic intravenous by the MiniCollect Capillary Blood Collection (0.9–1.1 mm inner diameter, 100 mm length). Euthanize the mice by cervical vertebra dislocation.
  b) Store the blood in 4°C for at least 12 h, and then centrifuge in 4°C, 2500 × *g* for 30 min.
  c) Carefully collect the supernatant and then centrifuge in 4°C, 13000 × *g* for 5 min. Collet the serum and store in 4°C for immediately usage or −80°C for longer storage.
  d) Resuspend 1 × 10^8^ *C. rodentium* with 100 μL PBS buffer with proteinase inhibitor. Dissociate *C. rodentium* by ultrasonication on the ice for 3 times, 1 minute per time. Add 300 μL B-PER Complete Bacterial Protein Extraction Reagent.
  e) Mix with gentle vortex for 30 min at room temperature (20°C–25°C). Centrifuge in 4°C, 16000 × *g* for 20 min to remove the cell debris.
  f) Quantify total protein with the BCA protein assay.
  g) Coat *C. rodentium* antigens at 5 μg/mL in 100 μL coating buffer on 96-well plates at 4°C for at least 16 h, and then wash the plate with PBST buffer.
  h) Incubate the diluted serum on the plate and detect IgG using HRP-labeled antibodies (SouthernBiotech) following the instruction of the kit.
  i) Develop the plate with ABTS substrate (SouthernBiotech). Detect OD at 405 nm with an ELISA reader (BioTek).

**CRITICAL:** (1) To find out the mLNs, you can firstly find the cecum and search upstream along the mesenteries. The mLNs are always located in the central point of the mesenteries, umbraculiferously connecting the small intestine (as shown in Figure 7A). All the mLNs should be collected. (2) When washing the biotin-labeled antibody, wash thoroughly by FACS buffer to avoid unspecific staining.

## Expected outcomes

**Figure 5:**
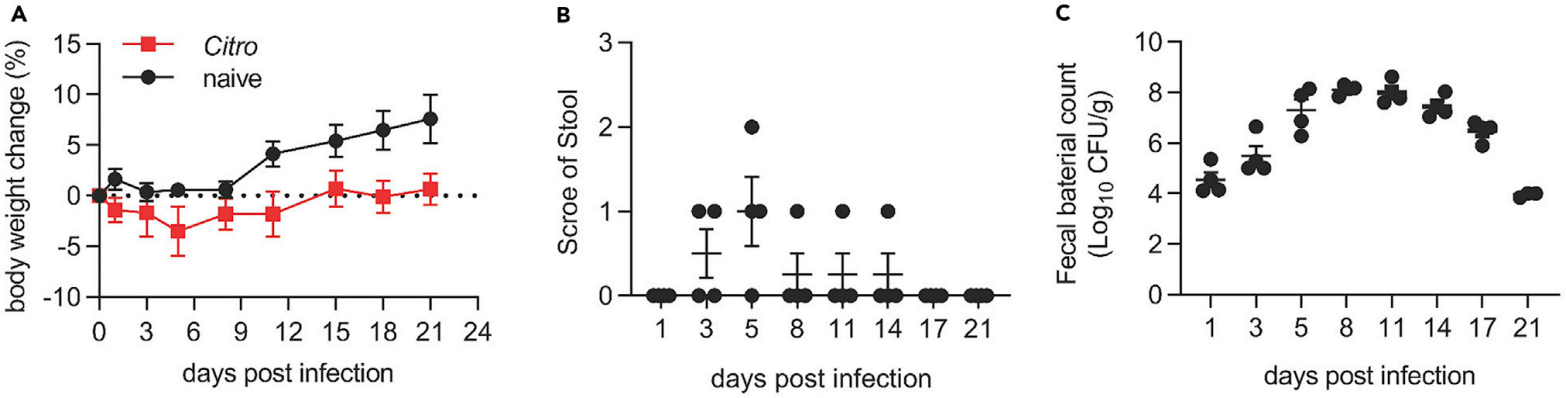
Mice post *C. rodentium* infection The body weight change (A), stool score (B), and fecal *C. rodentium* titers (C) post infection.

Using the above method, we observed that the weight of *C. rodentium* infected mice was decreased slightly in the first 5 days and then began to recover (Figure 5A). The stools became loose since the day 3–5, and then recovered later (Figure 5B). The *C. rodentium* burden in the feces peaked around day 8 post infection and then was began to be eliminated (Figure 5C).

**Figure 6:**
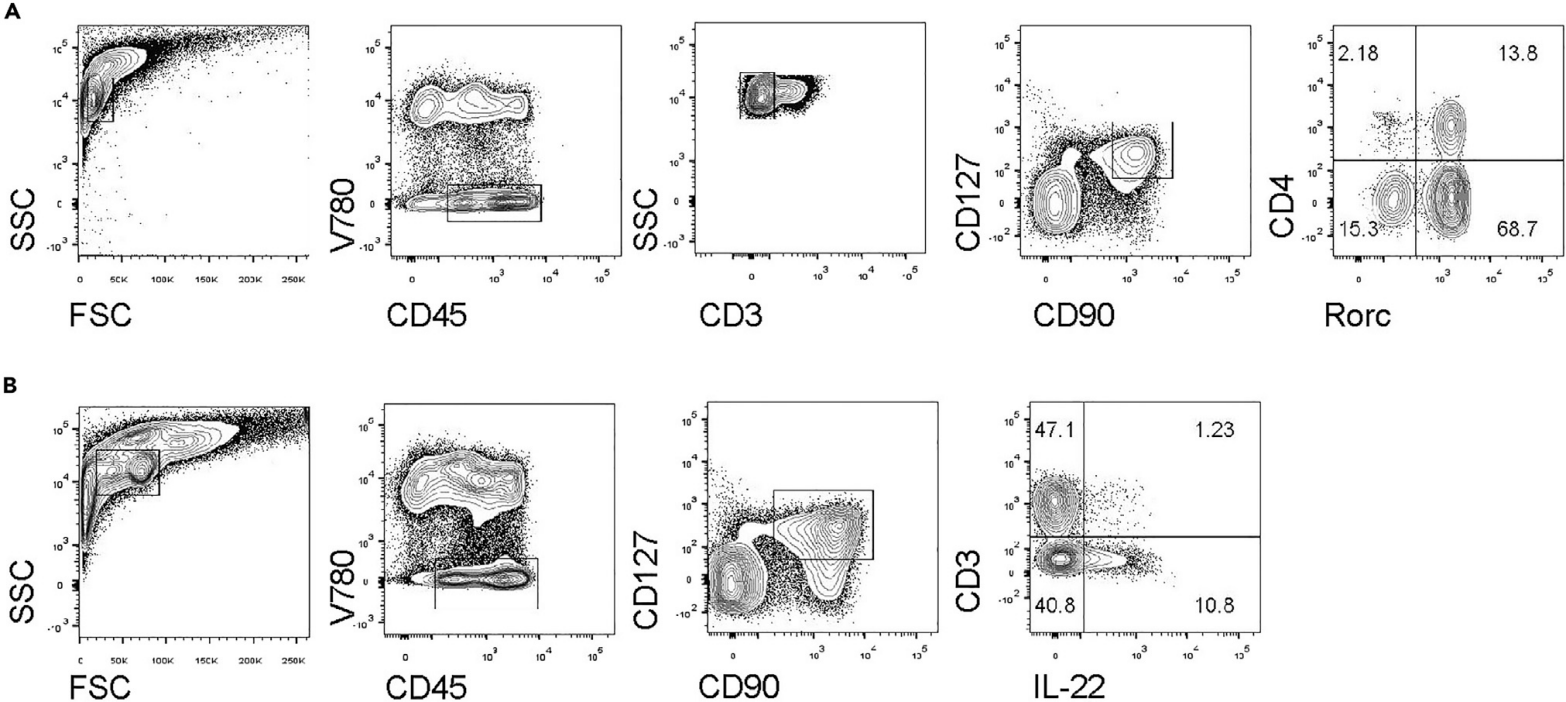
Gating strategy of ILC3s Gating strategy for the proportion of ILC3s (A) and function of ILC3s (B) in the colon.

At day 5 post infection, LPLs were isolated from the colon and ILC3s were analyzed by the flow cytometry. The ILC3s were gated as V780^−^CD45^+^CD90^+^CD3^−^CD127^+^RORγt^+^ (Guo et al., 2016; Wang et al., 2020), of CD4^+^ and CD4^−^ subsets (Figure 6A). The proportion of RORγt^+^ILC3s was almost 80% of ILCs in the colon. The IL-22^+^ ILC3s were gated in V780^−^CD45^low^CD90^+^CD3^−^CD127^+^ (Figure 6B). IL-22 is produced at the early stage of infection and is essential for the host defense against *C. rodentium* infection (Guo et al., 2014). The proportion of IL-22 producing ILC3s was about 30% in the gut with the pathogen infections.

**Figure 7:**
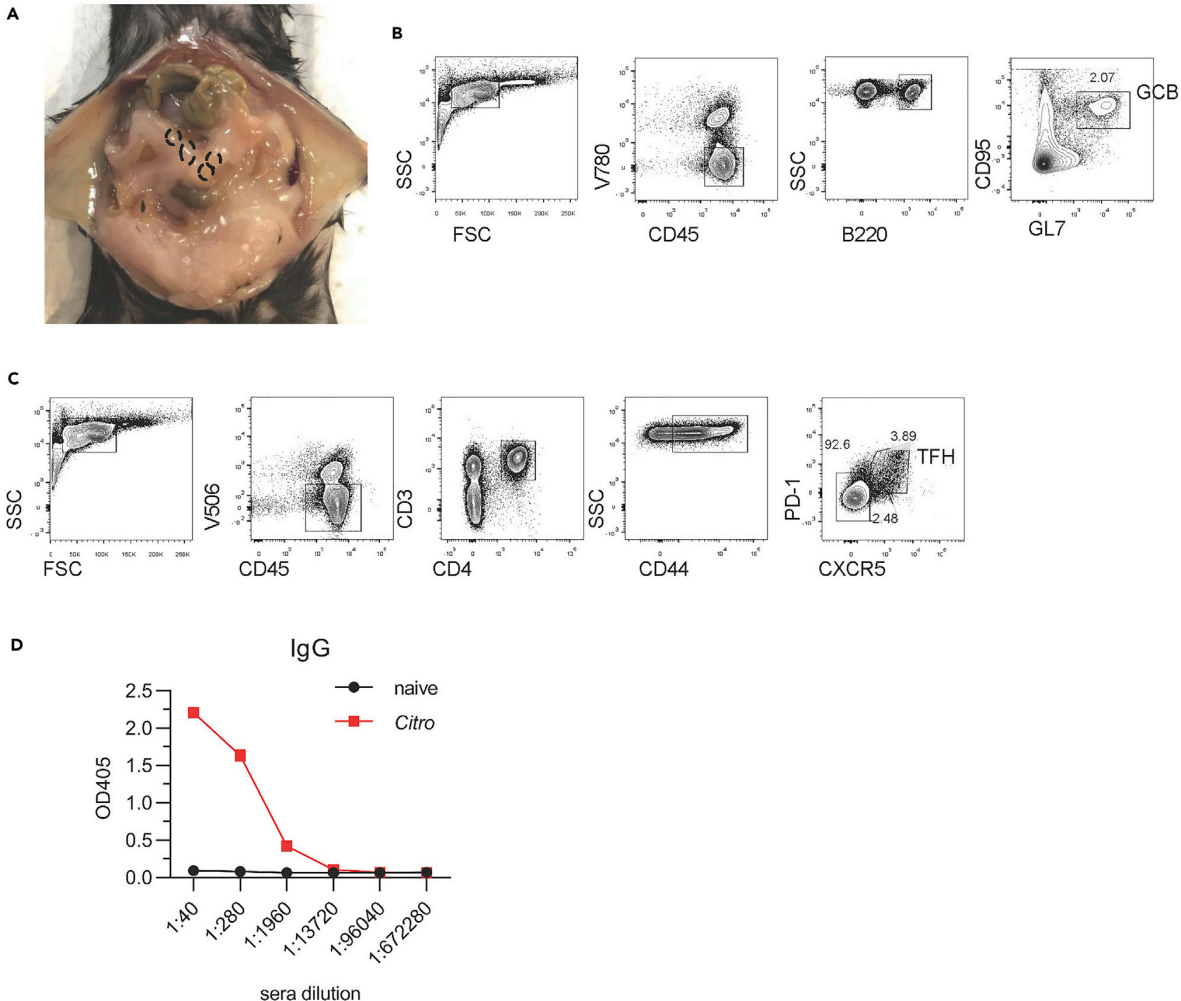
The adaptive immune response post infection (A) The location of mLN (black circle). (B) Gating strategy for GCB in the mLN. (C) Gating strategy for TFH in the mLN. (D) The *C. rodentium-*specific IgG production in the serum.

At day 10 post infection, the proportion of GCB and TFH were analyzed in the mLNs. The GCB was gated as V780^−^CD45^+^B220^+^CD95^+^GL7^+^ (Figure 7B) and the TFH was gated as V506^−^CD45^+^CD3^+^CD4^+^CD44^+^CXCR5^+^PD1^+^ (Figure 7C) (Wang et al., 2020; Melo-Gonzalez et al., 2019). The *C. rodentium*-specific IgG was elevated in the serum at day 21 post infection (Figure 7D).

## Limitations

The model of *C. rodentium* infection is dependent on the status of mice and microbiota. Before infection, it is better to host the mice in the same environment to settle down for at least 3 days. To avoid the influences from the microbiota due to different genotypes or treatments, different genotyped mice or mice undergoing different treatment should not be cohoused. Mice from different background will exhibit different susceptibility to the infection (Mondelaers et al., 2016), thus the researchers should choose the mice with the same genetic and raising background. Gender also influences the severity of the infection. Compared with female mice, male mice are more susceptible to weight change and easier for fecal collection.

The ingredient of diet is essential for the pathogen defense. High fiber diet could protect the mice from pathogen infections (Desai et al., 2016), while the high fat diet could aggravate the infection (Määttänen et al., 2018). Here we use the basic diet just for mice growth and reproduction.

A similar protocol has been published in 2013 on JOVE (Bhinder et al., 2013). Our protocol has enriched the details on the infection procedures and the assessment of immune responses rather than pathological changes in the model.

## Troubleshooting

### Problem 1

Mice die within 1 day post infection.

### Potential solution

In this low dose infection model, wild type mice would not die during the process. It is possible that improper oral gavage can cause fatal tracheal trauma to the mice. As in Figure 2B, the gavage feeding needle should insert to the stomach with no obstruction.

### Problem 2

Other bacteria grow on the plate.

### Potential solution

Since day 5 post infection, some other bacteria like *E. coli* could be observed on the plate. Researchers could distinguish between *E. coli* and *C. rodentium* by the color and size of colony. The colony of *C. rodentium* should be as shown in Figure 3B. According to the colony character of the stock *C. rodentium* on the plate, neither too pink or too purple of color, nor too big or too small of size should be counted as *C. rodentium* (as shown in Figure 3C, black arrows).

### Problem 3

Few LPLs isolated.

### Potential solution

If there are too many epithelial cells remaining after the digestion, which could entangle and prevent the centrifugation of LPLs from the cell suspension. Enlarge the volume of 40% and 80% Percoll and change the 5 mL tube to 15 mL tube for centrifugation. It will increase the area and lower the cell density to let the LPLs pass through.

### Problem 4

Low viability of LPLs.

### Potential solution

Store the tissues on the ice until the Wash Buffer I. Do not digest the tissue for longer time than the protocol states. After the digestion, neutralize the digestion buffer immediately with the 1640 buffer. After the Percoll centrifuge, wash the cells at least twice with FACS buffer to remove residual Percoll.

### Problem 5

No RORγt^+^ or IL-22^+^ staining, or the positive and negative subsets are not clearly separated.

### Potential solution

Equilibrate the fixation buffer and permeabilization buffer to room temperature (20°C–25°C) before use or fix the cells in 4°C for at least 12 h. Wash the cells thoroughly with permeabilization buffer to remove residual fixation buffer, which could impair the staining.

## Resource availability

### Lead contact

Further information and requests for resources and reagents should be directed to and will be fulfilled by the Lead Contact, Xiaohuan Guo (guoxiaohuan@tsinghua.edu.cn).

### Materials availability

This study did not generate unique reagents.

### Data and code availability

This study did not generate any unique datasets or code.
